# Differences in adherence to treatment, relapses and healthcare costs between delusional disorder and paranoid personality disorder

**DOI:** 10.1192/j.eurpsy.2024.298

**Published:** 2024-08-27

**Authors:** R. Álvarez-García, S. Abascal-Peiró, A. Gonzalo de Miguel, C. Blanco-Londono, A. Martinez-Pillado, L. Mata-Iturralde, E. Baca-Garcia

**Affiliations:** ^1^Hospital Universitario Rey Juan Carlos; ^2^Hospital Universitario Fundacion Jimenez Diaz, Madrid, Spain

## Abstract

**Introduction:**

Limited information is available regarding the clinical features, optimal treatment and prognosis of Paranoid Personality Disorder (PPD) and Delusional Disorder (DD). This is partly due to the low prevalence of cases and poor patient insight. The difference between DD and PPD has been questioned in the literature, as some studies have described them as a continuum, highlighting the role of specific personality traits in the transition to clinical delusions.

Nonadherence to pharmacological treatment is one of the most challenging aspects. This further leads to relapses, increased use of emergency psychiatric services, psychiatric admissions, longer periods of hospitalization, and an increased cost of illness to healthcare systems.

**Objectives:**

The primary goal of this study is to compare the differences between DD and PPD in terms of medication adherence, relapses, lost to follow-up, and costs. Other aims of this study are to analyze the differences in these variables between patients who are adequately adherent and patients who are not

**Methods:**

An observational, retrospective, and multicenter descriptive epidemiological study was conducted. Patients were selected from four public departments of psychiatry in Madrid, providing an area of roughly one million people. All patients were older than 18 years-old, diagnosed with DD or PPD from 2005 to 2022. Data were extracted from electronic medical records and from electronic prescribing program used in the public health system.The study was approved by the Hospital Fundación Jiménez Díaz Ethics Committee.

**Results:**

1227 individuals diagnosed with DD (974 patients,79,3%) or PPD (253 patients, 20.61%). 23.81% (232 patients) of the DD-group did not take out the prescribed medication of the pharmacy, and 16.6% (42 patients) of the PPD-group were considered non-adherent.

Adherent patients had greater follow-up (4.02 vs 2.89 years) and shorter hospital stays (5.15 vs 8.6 days, p<0.05) compared to non-adherent patients. DD patients doubled the average hospitalization stay compared to the PPD group (6.7 vs 2.96 days, p<0.01).

Regarding costs: DD had higher hospitalization costs than PDD (1164 vs 488 euros per year) and higher total costs than PDD (2180 vs 1528 euros per year, p<0.05). The costs were also higher in non-adherent than in adherent patients (2570 vs 1895 euros per year, p<0.05)

**Conclusions:**

Our sample of 1227 DD and PPD patients followed from 2005-2022 is, to our knowledge, one of the largest collected to date. We found sociodemographic and clinical differences between the DD and the PPD group. We also found differences between adherent and non-adherent patients, highlighting that non-adherence is associated with longer mean stay of hospitalization and more costs, both hospitalization and total direct healthcare costs. We have also found association between non-adherence and risk of psychotic relapse.

**Disclosure of Interest:**

None Declared

